# High Burden of Intestinal Colonization With Antimicrobial-Resistant Bacteria in Chile: An Antibiotic Resistance in Communities and Hospitals (ARCH) Study

**DOI:** 10.1093/cid/ciad283

**Published:** 2023-07-05

**Authors:** Rafael Araos, Rachel M Smith, Ashley Styczynski, Felipe Sánchez, Johanna Acevedo, Lea Maureira, Catalina Paredes, Maite González, Lina Rivas, Maria Spencer-Sandino, Anne Peters, Ayesha Khan, Dino Sepulveda, Loreto Rojas Wettig, María Luisa Rioseco, Pedro Usedo, Pamela Rojas Soto, Laura Andrea Huidobro, Catterina Ferreccio, Benjamin J Park, Eduardo Undurraga, Erika M C D’Agata, Alejandro Jara, Jose M Munita

**Affiliations:** Instituto de Ciencias e Innovación en Medicina, Facultad de Medicina Clínica Alemana Universidad del Desarrollo, Santiago, Chile; Advanced Center for Chronic Diseases (ACCDiS), Santiago, Chile; Multidisciplinary Initiative for Collaborative Research in Bacterial Resistance (MICROB-R), Santiago, Chile; US Centers for Disease Control and Prevention, Atlanta, Georgia, USA; US Centers for Disease Control and Prevention, Atlanta, Georgia, USA; Instituto de Sociología, Pontificia Universidad Católica de Chile, Santiago, Chile; Instituto de Ciencias e Innovación en Medicina, Facultad de Medicina Clínica Alemana Universidad del Desarrollo, Santiago, Chile; Centro de Control y Prevención del Cáncer (CECAN) FONDAP 152220002, Santiago, Chile; Instituto de Ciencias e Innovación en Medicina, Facultad de Medicina Clínica Alemana Universidad del Desarrollo, Santiago, Chile; Instituto de Ciencias e Innovación en Medicina, Facultad de Medicina Clínica Alemana Universidad del Desarrollo, Santiago, Chile; Instituto de Ciencias e Innovación en Medicina, Facultad de Medicina Clínica Alemana Universidad del Desarrollo, Santiago, Chile; Instituto de Ciencias e Innovación en Medicina, Facultad de Medicina Clínica Alemana Universidad del Desarrollo, Santiago, Chile; Multidisciplinary Initiative for Collaborative Research in Bacterial Resistance (MICROB-R), Santiago, Chile; Instituto de Ciencias e Innovación en Medicina, Facultad de Medicina Clínica Alemana Universidad del Desarrollo, Santiago, Chile; Multidisciplinary Initiative for Collaborative Research in Bacterial Resistance (MICROB-R), Santiago, Chile; Instituto de Ciencias e Innovación en Medicina, Facultad de Medicina Clínica Alemana Universidad del Desarrollo, Santiago, Chile; Multidisciplinary Initiative for Collaborative Research in Bacterial Resistance (MICROB-R), Santiago, Chile; Department of Pathology, Microbiology and Immunology, Vanderbilt University Medical Center, Nashville, Tennessee, USA; Instituto de Ciencias e Innovación en Medicina, Facultad de Medicina Clínica Alemana Universidad del Desarrollo, Santiago, Chile; Hospital Puerto Montt, Facultad de Medicina, Universidad San Sebastián, Chile; Hospital Puerto Montt, Facultad de Medicina, Universidad San Sebastián, Chile; Hospital Regional de Antofagasta, Universidad de Antofagasta, Chile; Laboratorio Clínico, Hospital Padre Hurtado, Chile; Departamento de Ciencias Preclínicas, Facultad de Medicina, Universidad Católica del Maule, Talca, Chile; Advanced Center for Chronic Diseases (ACCDiS), Santiago, Chile; Facultad de Medicina, Departamento de Salud Pública, Pontificia Universidad Católica de Chile, Santiago, Chile; US Centers for Disease Control and Prevention, Atlanta, Georgia, USA; Multidisciplinary Initiative for Collaborative Research in Bacterial Resistance (MICROB-R), Santiago, Chile; Escuela de Gobierno, Pontificia Universidad Católica de Chile, Santiago, Chile; Research Center for Integrated Disaster Risk Management (CIGIDEN), Santiago, Chile; CIFAR Azrieli Global Scholars program, CIFAR, Toronto, Canada; Division of Infectious Diseases, Warren Alpert School of Medicine, Brown University, Providence, Rhode Island, USA; Facultad de Matemáticas, Pontificia Universidad Católica de Chile, Santiago, Chile; Instituto de Ciencias e Innovación en Medicina, Facultad de Medicina Clínica Alemana Universidad del Desarrollo, Santiago, Chile; Multidisciplinary Initiative for Collaborative Research in Bacterial Resistance (MICROB-R), Santiago, Chile

**Keywords:** antimicrobial-resistance, colonization, gram-negative, community, Latin America

## Abstract

**Background:**

Antimicrobial resistance is a global threat, heavily impacting low- and middle-income countries. This study estimated antimicrobial-resistant gram-negative bacteria (GNB) fecal colonization prevalence in hospitalized and community-dwelling adults in Chile before the coronavirus disease 2019 pandemic.

**Methods:**

From December 2018 to May 2019, we enrolled hospitalized adults in 4 public hospitals and community dwellers from central Chile, who provided fecal specimens and epidemiological information. Samples were plated onto MacConkey agar with ciprofloxacin or ceftazidime added. All recovered morphotypes were identified and characterized according to the following phenotypes: fluoroquinolone-resistant (FQR), extended-spectrum cephalosporin-resistant (ESCR), carbapenem-resistant (CR), or multidrug-resistant (MDR; as per Centers for Disease Control and Prevention criteria) GNB. Categories were not mutually exclusive.

**Results:**

A total of 775 hospitalized adults and 357 community dwellers were enrolled. Among hospitalized subjects, the prevalence of colonization with FQR, ESCR, CR, or MDR-GNB was 46.4% (95% confidence interval [CI], 42.9–50.0), 41.2% (95% CI, 37.7–44.6), 14.5% (95% CI, 12.0–16.9), and 26.3% (95% CI, 23.2–29.4). In the community, the prevalence of FQR, ESCR, CR, and MDR-GNB colonization was 39.5% (95% CI, 34.4–44.6), 28.9% (95% CI, 24.2–33.6), 5.6% (95% CI, 3.2–8.0), and 4.8% (95% CI, 2.6–7.0), respectively.

**Conclusions:**

A high burden of antimicrobial-resistant GNB colonization was observed in this sample of hospitalized and community-dwelling adults, suggesting that the community is a relevant source of antibiotic resistance. Efforts are needed to understand the relatedness between resistant strains circulating in the community and hospitals.

## Teaser

We report a high colonization burden resulting from antimicrobial-resistant Gram-negative bacteria in hospitals and a community in Chile. Strikingly, 29% (95% confidence interval, 24–34) of community-dwelling adults carried extended-spectrum cephalosporin-resistant Enterobacterales, highlighting the magnitude of the community reservoir of antimicrobial resistance.

Antimicrobial resistance (AMR) is a global health threat. Despite prevention and control efforts, AMR's burden continues to increase, particularly in low- and middle-income countries. Global estimates suggest that nearly 5 million deaths were associated with AMR in 2019, and costs could reach US$1 to 2 trillion annually if not addressed [1, 2]. Antimicrobial-resistant gram-negative bacteria (AR-GNB), such as multidrug-resistant (MDR) *Escherichia coli*, *Klebsiella pneumoniae*, carbapenem-resistant Enterobacterales, carbapenem-resistant *Pseudomonas aeruginosa*, and *Acinetobacter baumannii* are of particular concern because they frequently produce difficult to treat or untreatable infections [3–6]. Low- and middle-income countries bear the most significant burden of AR-GNB, but there is a paucity of epidemiological data from such settings [1].

Reliable data on resistant pathogens are essential to implement infection control and antimicrobial stewardship efforts and inform research priorities. AR-GNB burden varies widely geographically, temporally, and even across healthcare settings within the same region. Current initiatives that seek to build AMR surveillance capacity, such as the World Health Organization's Global Antimicrobial Resistance and Use Surveillance System, focus on capturing incident clinical infections [7]. Although such an approach has long been the standard, it depends on substantial healthcare utilization and diagnostic stewardship capacities that are only sometimes available in low- and middle-income countries, which can introduce substantial ascertainment bias into surveillance data, resulting in inaccurate estimates [8, 9]. Additionally, effective surveillance of AR-GNB to support public health action needs to go beyond clinical isolates identified in healthcare settings and include assessments of the burden of AMR in communities. For most clinically meaningful AR-GNB, such as carbapenem-resistant Enterobacterales and extended-spectrum beta-lactamase (ESBL) producing Enterobacterales, colonization of the gastrointestinal tract is a well-characterized state, along with microbiota disruption and mucosal compromise, that precedes the development of AR-GNB clinical infections [10–14]. Thus, colonization may better represent the overall burden of AMR than infection.

In this study, we aimed to determine the burden of AR-GNB colonization in hospitals and a community. For this, we conducted point prevalence studies of hospitalized patients from 4 tertiary care hospitals in Chile and a population-based community cohort [15] predominantly served by 1 of these hospitals.

## METHODS

### Study Design and Sample

This study was part of the Antibiotic Resistance in Communities and Hospitals studies evaluating antimicrobial resistant bacteria (ARB) colonization prevalence in 6 countries [9].

We performed cross-sectional studies between December 2018 and May 2019 to determine the phenotypic and genotypic prevalence of intestinal colonization with AR-GNB among hospitalized and community-dwelling adults. For 5 consecutive days, we approached all hospital patients aged 16 years or older for recruitment from 4 tertiary-care public hospitals serving cities across the Chilean territory: Antofagasta, Santiago, Curico, and Puerto Montt. Local teams, comprising 5 nurses or physicians, performed daily rounds in each hospital. If a patient was unavailable, the team revisited the next day. For each hospital, we recorded the total number of eligible subjects, the number of subjects approached, and the total number of individuals who agreed to participate in the survey. Patients with acute diarrhea, gastrointestinal bleeding, or conditions precluding rectal sampling (eg, having colostomy bags) were excluded. The sample size for the hospital point prevalence studies was estimated to find a prevalence of colonization resulting from MDR-GNB at least 5% different from a theoretical 20% prevalence. Using an alpha of 0.05 and a power of 80%, we needed to recruit 528 subjects. Assuming a conservative 50% rejection rate, we aimed to approach at least 1056 subjects to achieve the target sample size.

A random sample of community-dwelling adults was recruited from a population-based community cohort designated MAUCO (Maule Cohort). MAUCO is an ongoing cohort following approximately 10 000 adults (aged 38–74 years) living in Molina, a semirural, agricultural town in central Chile [15, 16]. To appropriately capture the general composition of the cohort, our sample was stratified by age, sex, and rurality. MAUCO participants with fever, diarrhea, or respiratory symptoms at the time of recruitment were excluded from the study. Assuming a 10% prevalence of intestinal colonization resulting from AR-GNB, the sample size necessary to find a 5% difference in the proportion of colonized individuals was 316. We anticipated a 50% rejection rate; our target sample size was 632 individuals.

### Data Collection and Processing

Data were collected from participating hospitals using a standardized questionnaire. Similarly, standardized questionnaires were administered to all hospitalized and community participants to obtain demographic and relevant clinical data. Medical records were accessed to obtain additional details if needed.

Intestinal colonization was assessed using rectal swabs obtained by study personnel in the hospital or self-collected stool samples in the community. The methodologies for sampling, transporting, and processing study specimens have been published [13, 17, 18]. In brief, after specimens were collected either by hospital personnel or self-collection, they were transported to a local laboratory within 2 (hospital) and 48 (community) hours from collection. Specimens were then plated onto 2 screening plates of MacConkey agar supplemented with either ciprofloxacin (2 µg/mL) or ceftazidime (2 µg/mL), incubated for 24 hours, and shipped with dry ice to a central laboratory at Universidad del Desarrollo, Santiago, Chile, for further characterization. At receipt, incubated plates were immediately assessed for bacterial growth. All distinct morphotypes observed on each plate were subcultured and underwent species identification via matrix-assisted laser desorption ionization-time of flight. Antibiotic susceptibility testing was performed using the disk diffusion method as per CLSI 2019 recommendations [19]. All isolates exhibiting resistance to at least 1 extended-spectrum cephalosporin (ESC) (ie, ceftriaxone, cefotaxime, ceftazidime, or cefepime for Enterobacterales, and ceftazidime or cefepime for nonlactose fermenting gram-negative bacilli) underwent conventional, in-house polymerase-chain-reaction (PCR) testing for *bla*_TEM_, *bla*_SHV_, and *bla*_CTX-M_ genes. All isolates found to be resistant to 1 or more carbapenems (ie, meropenem, imipenem, or ertapenem for Enterobacterales, and meropenem or imipenem for nonlactose fermenting gram-negative bacilli) were phenotypically screened for carbapenemase production using Blue-Carba. Isolates with positive Blue-Carba test underwent molecular testing for *bla*_KPC_, *bla*_NDM_, *bla*_VIM_, *bla*_IMP_, and *bla*_OXA-48_. In addition, all isolates were studied for the presence of the *mcr*-1 gene by a previously standardized pooled PCR method in batches of 20 samples. Positive pools were deconvoluted, and individual PCR testing was performed to identify the specific isolates carrying the *mcr*-1 gene. Phenotypic susceptibility to colistin was not evaluated (Supplementary Figure 1).

### Data Analysis

The demographic information of hospitalized and community-dwelling adults was analyzed using descriptive statistics. We estimated the prevalence (95% confidence interval [CI]) of colonization with AR-GNB in both the hospital and community settings as the total number of individuals colonized with at least 1 AR-GNB over the total number of individuals enrolled in the study. The outcomes corresponded to 4 nonmutually exclusive phenotypical categories of AR-GNB: (1) fluoroquinolone resistant (FQR); (2) extended-spectrum cephalosporins resistant (ESCR); (3) carbapenem resistant (CR); and (4) multidrug resistant as per the Centers for Disease Control and Prevension definition [20].

The proportion of AR-GNB–carrying genes encoding beta-lactamases or carbapenemases was also analyzed according to the isolate's phenotype (Supplementary Figure 1). Prevalence data are presented at the participant level.

Analyses were performed using STATA/SE version 17.0 and R version 4.0.5.

### Ethics

All enrolled participants provided written informed consent. Our study was approved by the Research Ethics Committee CEC-MEDUC, Pontificia Universidad Católica de Chile, ID: 181105003.

## RESULTS

### Characteristics of Study Subjects

*Hospitalized patients.* A total of 1294 hospitalized adults were approached for study participation, of whom 775 (59.9%) agreed to participate, with the following distribution per facility: (1) Hospital Antofagasta (n = 225); (2) Hospital Santiago (n = 216); (3) Hospital Curico (n = 166); and (4) Hospital Puerto Montt (n = 168). The median (interquartile range) age was 60 (42–72) years, and 402 subjects (52%) were female. A total of 79% of patients were admitted to medical-surgical wards, 13% to maternity wards, and 9% to a critical care unit. The overall median Charlson comorbidity score was 3 (range, 0–5). A summary of the characteristics of the hospitalized participants by hospital is provided in Table 1.

**Table 1.** General Characteristics of Hospitalized Patients by Study Hospital

Variable	Antofagasta Hospital	Santiago Hospital	Curico Hospital	Puerto Montt Hospital	*P* Value
Sample (n)	225	216	166	168	
Age (y)^a^	59.9 (41.6–68.3)	62.1 (43.3–73.7)	60.1 (38.3–74.2)	57.2 (43.4–68.1)	.22
Female^b^	119 (52.9)	110 (50.9)	97 (58.4)	76 (45.2)	.11
Unit of hospitalization					
Medical/surgical^b^	171 (76.0)	185 (85.7)	123 (74.1)	133 (79.2)	.03
Maternity/gynecology^b^	31 (13.8)	22 (10.2)	28 (16.9)	16 (9.5)	
ICU^b^	23 (10.2)	9 (4.2)	15 (9.0)	19 (11.3)	
Charlson Index^a^ [21]	3 (0–4)	3 (0–5)	3 (0–4)	3 (.5–5)	.32
Surgery during hospitalization^b^	115 (51.3)	68 (31.6)	45 (27.4)	77 (46.7)	<.01
Use of central venous catheter in the current hospitalization^b^	50 (22.3)	23 (10.7)	12 (7.2)	24 (14.6)	<.01
Use of urinary catheter at the day of specimen collection^b^	29 (13.6)	25 (11.9)	22 (13.3)	44 (26.8)	<.01
Invasive mechanical ventilation during hospitalization (current or past)^b^	26 (11.6)	30 (13.9)	12 (7.3)	11 (6.8)	.06
Prior MDR-GNB colonization (based on local laboratory data)^b^	48 (21.3)	29 (13.4)	3 (1.8)	10 (6.0)	<.01
Length of stay (admission to swab)^a^	4 (1–12)	4 (1–10)	6 (3–12)	7 (4–17)	<.01
Antimicrobial exposure prior to study participation (14 d before rectal swab)^b^	38 (21.7)	18 (10.0)	7 (4.3)	18 (12.0)	<.01
Current antimicrobial exposure	142 (63.1)	126 (59.7)	85 (51.8)	107 (64.5)	<.01
Current use of PPI	175 (77.8)	147 (68.1)	119 (71.7)	80 (47.6)	<.01

December 2018 to May 2019.

Values are shown as ^a^median (interquartile range) or ^b^number (percentage). *P*-values from χ^2^ or Kruskal Wallis test for categorical or continuous variables, respectively. Surgery during hospitalization and use of central venous catheter in the current hospitalization refer to any surgical intervention taking place between hospital admission and the time of the rectal swab sampling. Invasive mechanical ventilation (IMV) during hospitalization at the time of the rectal swab is labeled as “current” or “past” if the participants underwent IMV before the obtention of the rectal swab. Previous MDR-GNB colonization refers to participants that have a history of MDR-GNB colonization based on laboratory information available in the participant's medical record up to 1 year before study participation.

Abbreviations: ICU, intensive care unit; MDR-GNB, multidrug-resistant gram-negative bacteria; PPI, proton pump inhibitor.

*Community-dwelling individuals.* A total of 601 community-dwelling adults participating in the MAUCO cohort were approached, of whom 357 (59.4%) were enrolled. The median (interquartile range) age was 55 (48–62) years, and 224 subjects (62.8%) were female. Exposure to the inpatient healthcare setting was low as 21 subjects (5.0%) recalled undergoing hospitalization in the previous 6 months. In contrast, 244 (68.4%) participants reported at least 1 visit to the local emergency department (ED) or an ambulatory clinic during the prior 6 months. Fifty-seven (16.3%) subjects reported using antimicrobials in the past 3 months, of whom 45 (78.9%) had a history of ED or ambulatory clinic visits. Table 2 summarizes the most relevant epidemiological and clinical characteristics of the MAUCO sample.

**Table 2.** General Characteristics of Community-Dwelling Adults From the Maule Cohort (MAUCO), December 2018 to May 2019

Variable	MAUCO
Sample (n)	357
Age^a^	55 (48–62)
Female^b^	224 (62.8)
Co-residence (persons per household)^a^	3 (2–4)
Hospitalization (6 mo)^b^	18 (5.1)
ED or ambulatory clinic visits (6 mo)^b^	224 (68.4)
BMI (kg/m^2^)^c^	29.5 (26.3–32.7)
Diabetes^b^	54 (15.4)
Liver disease^b^	82 (23.2)
Chronic renal disease^b^	8 (2.2)
Cancer^b^	8 (2.3)
Cardiovascular disease^b^	129 (36.5)
Respiratory disease^b^	12 (3.4)
Diarrhea (6 mo)^b^	65 (18.3)
Antibiotic exposure (3 mo)^b^	57 (16.3)
Antibiotic exposure Household (3 mo)^b^	32 (9.5)

Values shown as ^a^median (interquartile range), ^b^number (percentage), or ^c^mean (standard deviation).

Abbreviations: Amb, ambulatory; BMI, body mass index; ED, emergency department; MAUCO, Maule cohort.

### Prevalence of AR-GNB Colonization at the Hospital and Community Level

Among the 775 hospitalized patients, 434 (56%) exhibited bacterial growth in at least 1 positive screening plate. A total of 1136 morphotypes were identified and confirmed phenotypically as part of the previously mentioned AR-GNB categories. Supplementary Figure 2 shows the distribution of the most prevalent AR-GNB species and their main antimicrobial susceptibility patterns. Strains of *E. coli*, *K. pneumoniae*, and *Enterobacter* spp. predominated.

Among hospital participants, the overall prevalence of gut colonization with FQR, ESCR, CR-GNB, and MDR-GNB was 46.4% (95% CI, 42.9–50.0), 41.2% (95% CI, 37.7–44.6), 14.5% (95% CI, 12.0–16.9), and 26.3% (95% CI 23.2–29.4), respectively. The prevalence of colonization varied by the hospital, with patients from Hospital Antofagasta demonstrating the highest prevalence of FQR, ESCR, and MDR organisms (57%, 51%, and 37%, respectively), whereas participants from Hospital Curico exhibited the highest prevalence of CR-GNB (19%) colonization (Table 3). The most prevalent antimicrobial-resistant genes (ARG) carried by isolated AR-GNBs with phenotypic resistance to ESC or carbapenems are summarized in Table 4. A total of 280 hospital participants were colonized with at least 1 ESCR-GNB carrying 1 or more genes encoding for beta-lactamases, representing 87.8% of all participants colonized by AR-GNB with an ESCR phenotype. In contrast, the proportion of patients carrying carbapenemase-producing CR-GNB was 21.4% among participants colonized with GNB phenotypically resistant to carbapenems.

**Table 3.** Prevalence of AR-GNB Colonization in the Hospital and Community Settings

Gram-negative bacteria resistant to:	Antofagasta Hospital N = 225	Santiago Hospital N = 216	Curico Hospital N = 166	Puerto Montt Hospital N = 168	MAUCO N = 357
Fluoroquinolones	128 (56.9%)	82 (38.0%)	83 (50.0%)	67 (39.9%)	141 (39.5%)
95% CI	50.4–63.4	31.5–44.4	42.4–57.6	32.5–47.3	34.4–44.6
Extended-spectrum cephalosporins	115 (51.1%)	77 (35.6%)	73 (44.0%)	54 (32.1%)	103 (28.9%)
95% CI	44.6–57.6	29.3–42.0	36.4–51.5	25.1–39.2	24.2–33.6
Carbapenems	24 (10.7%)	29 (13.4%)	32 (19.3%)	27 (16.1%)	20 (5.6%)
95% CI	6.6–14.7	8.9–18.0	13.3–25.3	10.5–21.6	3.2–8.0
Multidrug resistant^a^	78 (34.7%)	51 (23.6%)	41 (24.7%)	34 (20.2%)	17 (4.8%)
95% CI	28.4–40.9	17.9–29.3	18.1–31.3	14.2–26.3	2.6–7.0

Categories are not mutually exclusive.

Abbreviations: AR-GNB, antimicrobial-resistant gram-negative bacteria; CI, confidence interval; MAUCO, Maule cohort; NA, not available.

Multidrug-resistant bacteria were defined as resistance to at least 1 compound of ≥ 3 antibiotic classes. The differences in the prevalence of intestinal colonization with resistant gram-negative bacteria between hospitals were statistically significant (χ^2^, *P* < .01) for fluoroquinolones, extended-spectrum cephalosporins, and multidrug resistance, and were not statistically significant for carbapenem (*P* = .09).

**Table 4.** Participant Level Distribution of Antimicrobial Resistant Genes Carried by AR-GNB in the Hospital and Community Settings

	Antofagasta Hospital N = 225	Santiago Hospital N = 216	Curico Hospital N = 166	Puerto Montt Hospital N = 168	MAUCO (Community) N = 357	Total N = 1132
Participant level						
PT with ESBL phenotype	115 (51.1%)	77 (35.6%)	73 (44.0%)	54 (32.1%)	103 (28.9%)	422 (37.3%)
PT carrying ESBL genes	108 (94%)	66 (86%)	64 (88%)	42 (78%)	69 (67%)	350 (83%)
Prevalence (%)	48	31	39	25	19	…
TEM	46 (43%)	20 (30%)	24 (38%)	13 (31%)	19 (28%)	122 (35%)
SHV	32 (30%)	31 (47%)	23 (36%)	19 (45%)	6 (9%)	111 (32%)
CTX-M	103 (95%)	57 (86%)	60 (94%)	37 (88%)	62 (90%)	320 (91%)
PT with CR phenotype and Blue Carba (+)	7 (3.1%)	7 (3.2%)	8 (4.8%)	10 (6.0%)	1 (0.3%)	33 (2.9%)
PT with *CR* gene^a^	6 (85.7%)	5 (71.4%)	8 (100%)	10 (100%)	0	24 (21%)
KPC	3 (50%)	5 (100%)	0	0	0	8| (33%)
NDM	0	5 (100%)	0	0	0	5 (21%)
IMP	0	0	0	0	0	0
VIM	3 (50%)	0	8 (100%)	9 (100%)	0	16 (67%)
OXA	0	0	0	0	0	0
PT with *mcr*-1	0	0	0	1	0	1

PT with ESBL phenotype denotes a participant colonized with at least 1 AR-GNB resistant to at least 1 extended-spectrum cephalosporin. PT carrying ESBL genes denotes a participant colonized with extended-spectrum cephalosporin-resistant organisms carrying at least 1 of the antimicrobial-resistant genes described in the table. PT with CR phenotype/Blue Carba (+) denotes a participant carrying at least 1 carbapenem-resistant gram-negative bacteria that resulted positive in the carbapenemase screening test (Blue Carba). PT with *CR* gene denotes a participant colonized with a carbapenem-resistant organism carrying at least 1 of the carbapenem-resistance genes described in the table.

Abbreviations: AR-GNB, antimicrobial-resistant gram-negative bacteria; CR, carbapenem-resistant; CTX-M, Cefotaxime-Munich; ESBL, extended-spectrum beta-lactamase; IMP, Imipenemase; KPC, Klebsiella pneumoniae carbapenemase; NDM, New Delhi metallo-beta-lactamase; OXA, Oxacillinase; PT, participant; SHV, Sulfhydryl variable; TEM, Temoniera; VIM, Verona integron-encoded metallo-beta-lactamase.

The percentage is calculated as the number of participants carrying a *CR* gene over the total number of participants carrying an AR-GNB with CR phenotype and a positive Blue Carba test. PT with *mcr*-1 denotes a participant colonized with at least 1 GNB carrying *mcr*-1 gene.

In the community setting, specimens obtained from 232 (65%) of the 357 participants exhibited growth in 1 or more screening plates, accounting for 528 morphotypes. Species distributions are summarized in Supplementary Figure 2. *E. coli* was the most common colonizing species found in this setting. *K. pneumoniae* and *Enterobacter* spp. were rarely found. The prevalence of colonization among community dwellers resulting from FQR, ESCR, CR, and MDR-GNB was 39.5% (95% CI, 34.4–44.6), 28.9% (95% CI, 24.2–33.6), 5.6% (95% CI, 3.2–8.0), and 4.8% (95% CI, 2.6–7.0), respectively. Table 4 shows the most prevalent ARG found in colonizing AR-GNB. Interestingly, 69 (67%) individuals colonized with ESCR-GNB had at least 1 gene that potentially encodes for ESBLs. Of note, 62 (69%) were positive for a blaCTX-M gene. No carbapenemase-encoding genes were found in the community.

## DISCUSSION

Our study found a high burden of fecal AR-GNB colonization in Chilean hospitals and an agricultural community in central Chile. Overall, colonization was higher in hospitals, reaching 46%, 41%, and 15% for FQR, ESCR, and CR-GNB, respectively. Worryingly, 26% of hospitalized subjects carried an MDR-GNB. Among patients colonized with ESCR-GNB, carriage of ARGs that encode for ESBL was almost universal (88%) and highly transmissible CTX-M-type enzymes were predominant. On the other hand, carbapenem resistance resulting from carbapenemase production was less frequent. Circulation of *bla*_VIM_ was observed in 3 centers, 2 hospitals had circulation of AR-GNB carrying *bla*_KPC_ genes, and only 1 reported *bla*_NDM_-carrying organisms. This finding may suggest a recent importation of carbapenemase genes among Chilean hospitals. The transmission potential of carbapenemases highlights the need to reinforce infection control practices in Chilean hospitals to avoid further transmission of carbapenemases.

Several factors may explain the high burden of AMR colonization observed in the hospital setting. First, antimicrobials are well-known disruptors of the colonization resistance against bacterial pathogens and exert tremendous selective pressure on microbial communities, setting ideal conditions for AR-GNB to colonize the human gut successfully [22]. As shown in Table 1, in our study, in-hospital antimicrobial exposure was common and ranged from 52% to 65%, according to the study hospital. Further analyses considering patterns of antimicrobial use or differing infection control practices may explain the differences in the prevalence of specific AR-GNB found in each hospital. Second, the sample of hospitalized subjects was frequently exposed to surgical and other invasive procedures, which may have increased their risk of AR-GNB colonization through cross-transmission. Finally, nonantimicrobial drugs, such as proton-pump inhibitors, were heavily used; this family of drugs has been pointed out as a relevant risk factor for AR-GNB acquisition, even in the absence of antimicrobial exposure [23].

The prevalence of fecal AR-GNB colonization among community-dwelling adults was lower than in hospitalized patients; however, rates were strikingly high compared with older reports and similar to a recent study from Botswana that also found a significant reservoir of ESCR Enterobacterales in a community [24, 25]. In our study, 40% of community-dwelling participants carried an FQR-GNB, whereas 29% were colonized with an ESCR-GNB, and 5% had GNB classified as MDR-GNB. Importantly, 67% of subjects colonized with an ESCR-GNB carried a potential ESBL-encoding gene, which in 90% of the cases corresponded to *bla*_CTX-M_, similar to the hospital setting. Although the impact of ESCR-GNB colonization among community-dwelling adults remains unknown, this finding suggests that the community reservoir of AMR is tremendous and may be driving the epidemiology of AMR in hospitals. Resistance to carbapenems was low and not from carbapenemase production. Potential drivers for AMR in this community include contamination from environmental sources. Although Chile has wide access to potable water, and the sewage system is fully connected to water treatment plants [26], a recent article reported significant river water and vegetable contamination resulting from FQR- and ESCR-GNB near the MAUCO area, suggesting that the environment may be a key player in spreading AMR among MAUCO participants [27]. Another explanation is the exposure to different selective pressures. Chile requires a medical prescription for using antimicrobials in the outpatient setting. In this study, the rate of antimicrobial use among MAUCO participants was modest (16%). However, almost 70% of the sample visited the ED or an ambulatory clinic. Thus, recall bias may have underestimated antimicrobial exposure. More studies are needed to determine the role of environmental contamination and healthcare exposure in driving AMR in the community.

Our work has limitations. First, as an observational study, it is subject to selection bias. Patients may differ in their willingness to participate in research activities in the hospital setting according to their baseline health status, which may bias the AR-GNB colonization estimation. Because of data privacy constraints, we could not compare the population that agreed to participate or not; therefore, we cannot know the direction of this bias. To provide generalizable results, we included a large sample of hospitalized patients that achieved our prespecified target sample size. All hospitals were in different regions of the country and belonged to the public health system, which provides healthcare to ∼80% of the Chilean population. Similarly, in the community setting, study subjects may share behavioral features such as risk aversion and healthcare-seeking behavior differing from those rejecting participation. In addition, MAUCO includes subjects aged 38 years or older only, excluding the younger and healthier local population [15, 16]. Thus, we may have overestimated the overall burden of AR-GNB colonization in this community. In addition, it is worth mentioning that the Charlson Comorbidity Index may not be an accurate estimation of the burden of comorbidities in the hospitalized population.

Second, we screened for AR-GNB colonization using culture techniques, which may misclassify the colonization status of a subject because of a lower sensitivity compared with molecular approaches [28]. However, culturing techniques are still extensively used for identifying AR-GNB colonization for infection control in the hospital setting. They provide clinically useful information in predicting the risk of adverse outcomes (eg, subsequent bacteremia) and allow for the characterization of isolated organisms. Also, using well-standardized sample collection and culture methods, our results can be compared with others and repeated over time to estimate trends [9]. Finally, using ceftazidime-based selective media for ESCR-GNB screening may not detect some ceftriaxone-resistant, ceftazidime-susceptible GNB. Resistance to ESC in GNB usually includes quinolone resistance; therefore, we expect this potential sensitivity loss to be minor because we included a second plate embedded with ciprofloxacin [29, 30].

In conclusion, our study is one of the first to deliver a comprehensive analysis of intestinal ARB colonization within both hospital and community settings in Chile, establishing the foundation for future regional research aimed at developing targeted interventions and policies. We found a high burden of AR-GNB colonization. Almost half of the hospitalized individuals carried FQR- or ESCR-GNB, whereas 1 of every 3 to 4 patients harbored at least 1 MDR-GNB. Within the community, one-third of the population carried at least 1 ESCR-GNB, often with highly transmissible ESBL genes (eg, *bla*_CTX-M_). This community AR-GNB reservoir may play a critical role in emergence and spread of AMR, underlining the importance of incorporating community-focused strategies in the approach to controlling AMR. Further research is essential to elucidate the community's role in AMR dissemination and assess the risk of subsequent infection for colonized individuals, particularly in low- and middle-income countries.

## Supplementary Data

Supplementary materials are available at *Clinical Infectious Diseases* online. Consisting of data provided by the authors to benefit the reader, the posted materials are not copyedited and are the sole responsibility of the authors, so questions or comments should be addressed to the corresponding author.

## Supplementary Material

ciad283_Supplementary_Data

## Notes

***Author Contributions.*** All authors have read and approved the final article.

***Financial support.*** This research was partially supported by the US Centers for Disease Control and Prevention, BAA #75D30118C02925 and Cooperative Agreement #1NU3HCK0000012-01-00 to RA, Asociación Nacional de Investigación y Desarrollo (ANID), Fondo Nacional de Desarrollo Científico y Tecnológico FONDECYT Grant #1211947 to J. M. and #1220907 to A. J., and Millenium Science Initiative Program #NCN12_059 to A. J. Infrastructure was supported by FONDEQUIP EQM150093 and FONDEQUIP EQM190110.

***Supplement sponsorship.*** This article appears as part of the supplement “The Evolving Challenges of Antibiotic Resistance in Low- and Middle-Income Countries: Priorities and Solutions,” sponsored by the U.S. Centers for Disease Control and Prevention, and Health Security Partners.
