# Supplementary material for: High Burden of Intestinal Colonization With Antimicrobial-Resistant Bacteria in Chile: An Antibiotic Resistance in Communities and Hospitals (ARCH) Study
Source: Clin Infect Dis. 2023 Jul 5;77(Suppl 1):S75–81. doi: 10.1093/cid/ciad283 (PMC10321693; doi:10.1093/cid/ciad283)
Supplement: ciad283_Supplementary_Data [file ciad283_supplementary_data.docx]

**Supplementary Figures**


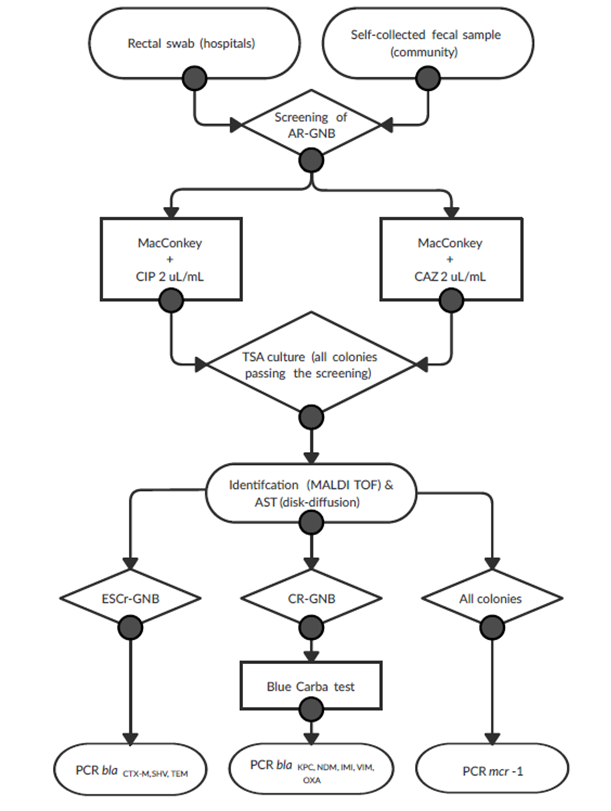


**Supplementary Figure 1.** Laboratory workflow. Stool samples collected at each site were screened for AR-GNB using selective media embedded with ciprofloxacin or ceftazidime. All colonies passing the screening plates and showing differential morphotypes were plated on TSA culture. Isolates were identified at the species level and antimicrobial susceptibility testing was done using the disk-diffusion method. The downstream molecular evaluation was based on the antimicrobial-resistance phenotypes, except for the case of *mcr*-1, whose presence was assessed in all isolates. AR-GNB: antimicrobial-resistant Gram-negative bacteria. CIP: ciprofloxacin. CAZ: ceftazidime. TSA: tryptic soy agar. AST: antimicrobial susceptibility testing. ESCr-GNB: extended-spectrum cephalosporin-resistant Gram-negative bacteria. CR-GNB: Carbapenem-resistant Gram-negative bacteria. PCR: polymerase-chain-reaction.

**A**

**
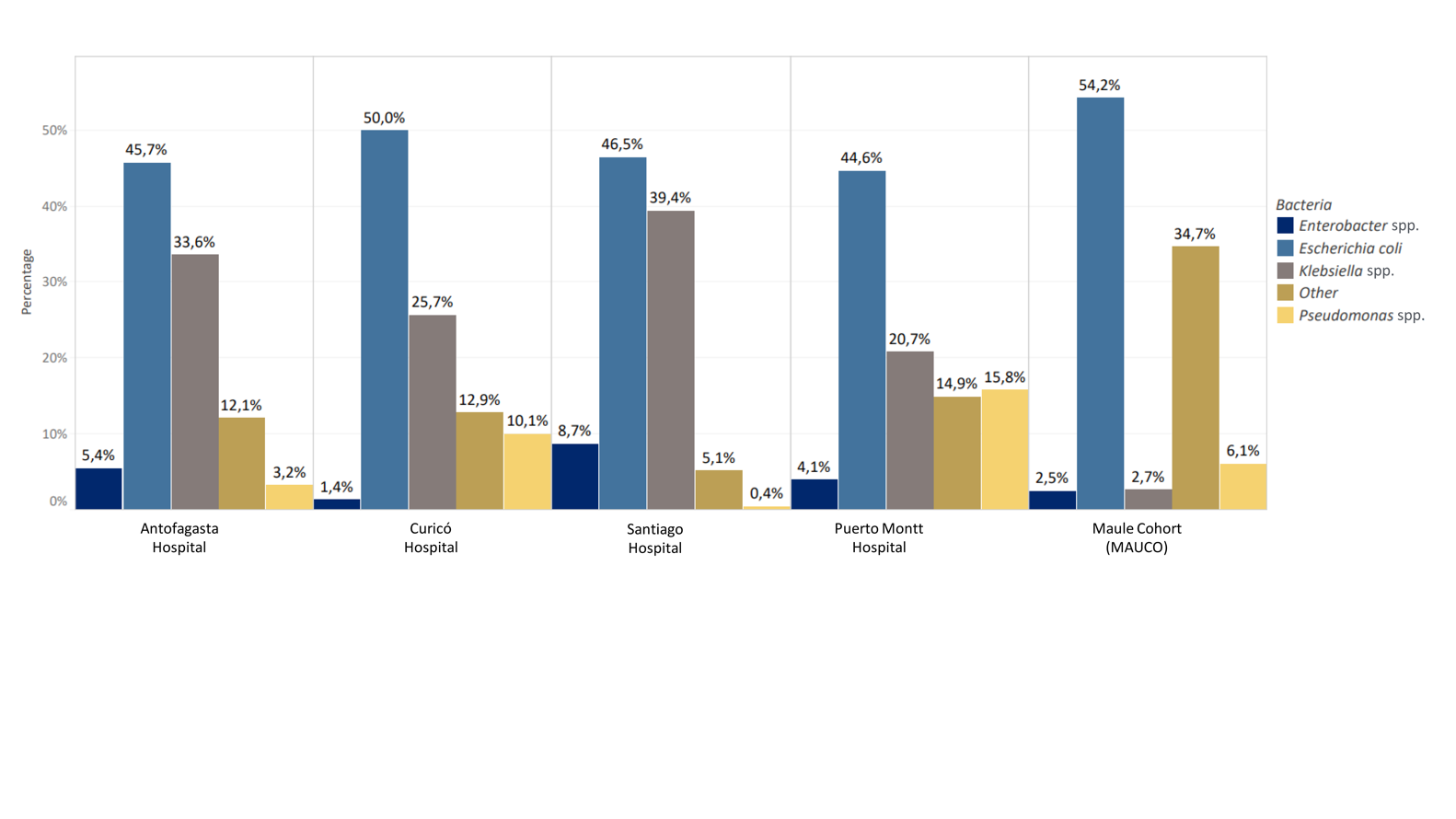
**

**B**

**
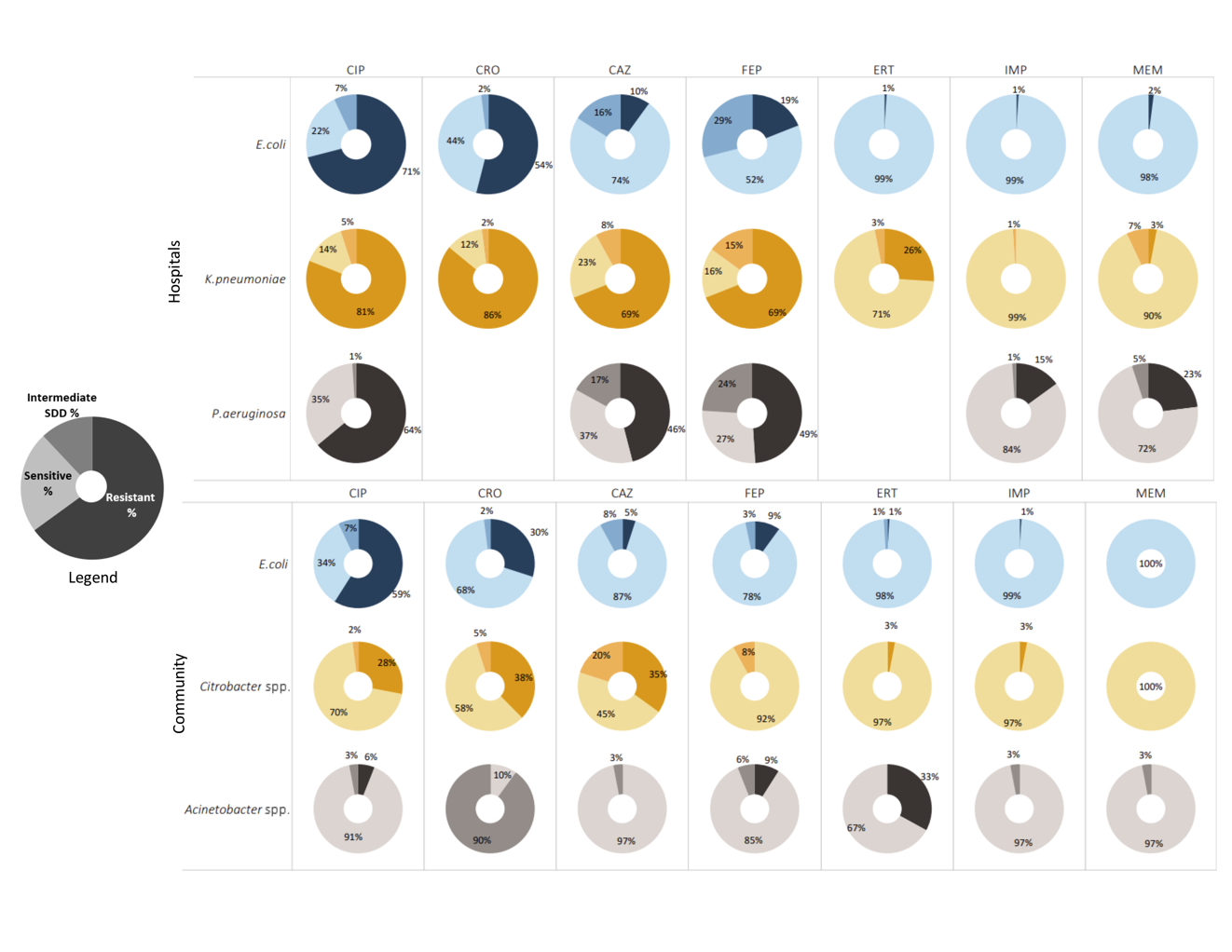
**

**Supplementary Figure 2**. The distribution of the most prevalent species of AR-GNB identified in hospitals and the community (A) and the overall susceptibility profile of the three most common colonizing species in hospitalized patients and the community (B). As a reference, each pie chart's dark, intermediate, and light colors (panel B) represent the percentage of resistant, intermediate (or susceptible dose-dependent), and susceptible isolates, respectively. CIP: ciprofloxacin; CRO: ceftriaxone; CAZ: ceftazidime; FEP: cefepime; ERT: ertapenem; IMP: imipenem; MEM: meropenem.
